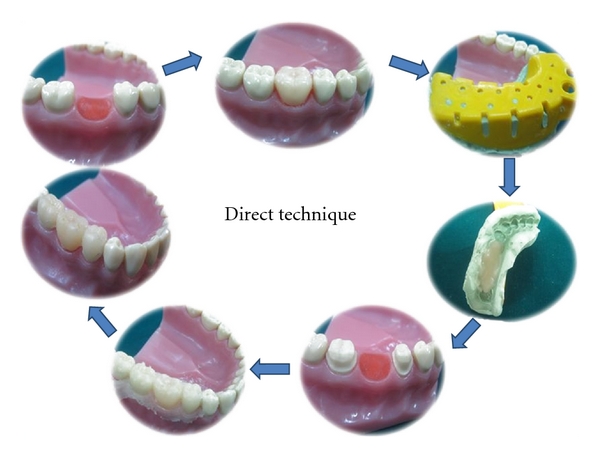# Techniques of Fabrication of Provisional Restoration: An Overview

**DOI:** 10.1155/2011/134659

**Published:** 2011-10-12

**Authors:** K. M. Regish, Deeksha Sharma, D. R. Prithviraj

**Affiliations:** Department of Prosthodontics, Govt. Dental College and Research Institute, Bangalore Victoria Hospital Campus, Fort 560002, Bangalore, India

## Abstract

A properly fabricated provisional restoration is important in achieving a successful indirect restoration. The importance of provisional restorations as an integral part of fixed prosthodontic treatment is evident from the abundance of the literature pertaining to their importance regarding margin fidelity, function, occlusion, and esthetics. There are a variety of techniques available to suit the individual needs of the clinician and of the clinical situation, from a single unit to a complete-arch provisional fixed prostheses.

## 1. Introduction

Fabrication of provisional restorations is an important procedure in fixed prosthodontics. Provisional restorations must satisfy the requirements of pulpal protection, positional stability, occlusal function, ability to be cleansed, margin accuracy, wear resistance, strength, and esthetics. They serve the critical function of providing a template for the final restorations once they have been evaluated intraorally [1].

Provisional restorations in fixed prosthodontic rehabilitation are important treatment procedures, particularly if the restorations are expected to function for extended periods of time or when additional therapy is required before completion of the rehabilitation [2].

Interim procedures also must be efficiently performed, because they are done while the patient is in the operatory and during the same appointment that the teeth are prepared. Costly chair side time must not be wasted, but the dentist must produce an acceptable restoration. Failure to do so results in the eventual loss of more time than was initially thought saved.

A well-made provisional fixed partial denture should provide a preview of the future prosthesis and enhance the health of the abutments and periodontium. The theories and techniques of fabrication for numerous types of provisional restorations abound in the dental literature [3].

Many procedures involving a wide variety of materials are available to make satisfactory interim restorations. As new materials are introduced, associated techniques are reported, and thus, there is even more variety. It is helpful principle that all the procedures have in common the formation of a mold cavity into which a plastic material is poured or packed.

Provisional restorations may be made directly on prepared teeth [4, 5] with the use of a matrix or indirectly by making an impression of the prepared teeth [6, 7]. A combination indirect-direct [8] technique is also possible which has evolved as a sequential application of these that involves fabrication of a preformed shell that is relined intraorally.

## 2. Search Strategy

A PubMed search of English literature was conducted up to January 2010 using the terms: provisional restorations, fixed partial denture, treatment restorations. Additionally, the bibliographies of 5 previous reviews as well as articles published in journal of prosthodontics, journal of prosthetic dentistry, general dentistry, and journal of american dental association were manually searched.

### 2.1. Indirect Provisional Fixed Partial Denture

The technique involves fabrication of the interim restoration outside the mouth. Fabrication of provisional restorations using the indirect technique eliminates the problems associated with the direct technique and also has the advantage that it can be partially delegated to auxiliary personnel [6]. Fisher et al. describes the use of an indirect technique for provisional fabrication that uses a fast-setting plaster. The technique has several advantages over the direct procedures. There is no contact of free monomer with the prepared teeth or gingival which might cause tissue damage and an allergic reaction or sensitization. The technique avoids subjecting prepared tooth to the heat evolved from the polymerizing resin. Indirect technique produces restoration with a superior marginal fit and as an auxiliary is involved in fabricating the restoration in the lab, it frees the patient and dentist for considerable amount of time [1, 9].

When compared to direct technique, it has fewer demerits. Principal disadvantage of the technique includes increased chair side time and increased number of intermediate steps. It is a tedious task to perform if there is inadequacy of assistants or the laboratory facilities. In addition, the technique involves use and possible damage of diagnostic casts [10].

**Procedure:**

**Figure 1:**
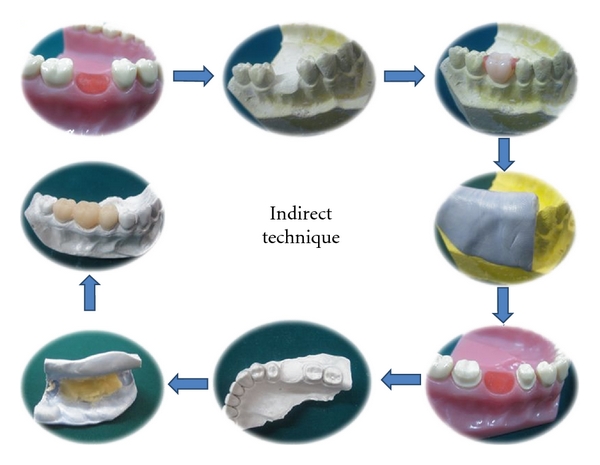


1. On the diagnostic cast, place a selected acrylic tooth on the area of the missing tooth, and seal it with the carding wax.
2. Following this, a silicone putty index is made involving at least one tooth each beyond the abutment teeth.
3. Prepare the patient's teeth in the usual manner.
4. Make a sectional impression of the prepared teeth and the adjacent structures and pour a check cast.
5. Lubricate the check cast with a petroleum jelly or any suitable separating media, mix the provisional restorative material, and place it in the tissue surface of the index and seat it on the check cast.
6. Try in the preformed restoration for its fit on the cast and intraorally.
7. Reline the temporary restoration to perfect the internal fit.
8. Finish, polish, and cement the restoration (Figure 1).

### 2.2. Indirect-Direct Provisional Fixed Partial Denture

The technique produces a custom made preformed external surface form of the restoration but the internal tissue surface form if formed by the underprepared diagnostic casts. This indirect-direct procedure has several advantages. With the combination indirect-direct technique, chair time can be reduced, since the provisional shell is fabricated before the patient's appointment. Enhanced control over restoration contours minimizes the time required for chair side adjustments. In addition, a smaller amount of acrylic resin will polymerize in contact with the prepared abutment, resulting in decreased heat generation, chemical exposure, and polymerization shrinkage compared to the direct technique [1]. Another advantage is that contact between resin monomer and soft tissues is reduced and less chances of allergic reactions.

The disadvantage of this procedure is the potential need of a laboratory phase before tooth preparation and the adjustments that are frequently needed to seat the shell completely on the prepared tooth.

**Procedure:**

**Figure 2:**
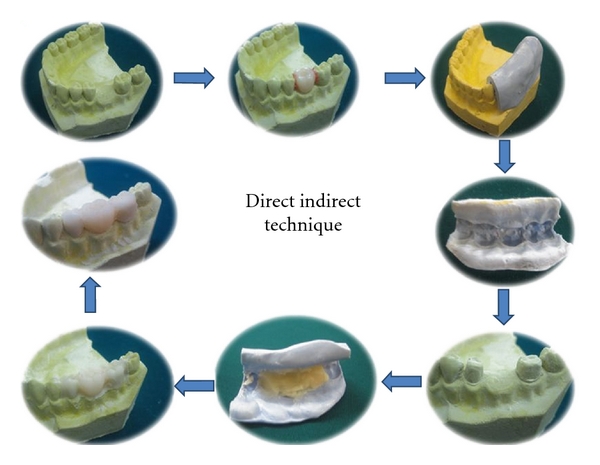


1. Pour an accurate pretreatment diagnostic cast from an impression of the unprepared teeth. For FPDs, wax a pontic into the edentulous area of the study cast, and modify with wax to obtain ideal contours, contacts, and occlusion.
2. Lightly lubricate the modified diagnostic cast, and make an impression using a high-viscosity elastomeric impression material. To provide an adequate bulk of material at the margins of the provisional, trim the sharp edge on the elastomeric over impression that represents the gingival crevice with a round bur to allow for extra bulk of resin material in this area. The silicone putty index is made involving at least on tooth each beyond the abutment teeth.
3. Remove the acrylic tooth and prepare the abutments on mounted diagnostic casts. (The diagnostic cast preparations should be more conservative than the eventual tooth preparation and should follow precisely the gingival margins.)
4. Lubricate the prepared diagnostic cast with a petroleum jelly or any suitable separating media, mix the provisional restorative material, and place it in the tissue surface of the index and reseat it on the prepared diagnostic casts.
5. After the acrylic resin has polymerized, finish the restoration. The provisional restoration should be paper thin and correctly contoured, and it should precisely follow the gingival margins on the cast.
6. Prepare the patient's teeth in the usual manner (to the gingival margins).
7. Try in the preformed restoration. (If the amount of tooth reduction is adequate, the provisional restoration will show optimal marginal fit with no need for adjustment.)
8. Reline the temporary restoration to perfect the internal fit.
9. Finish, polish, and cement the restoration (Figure 2) [6, 8].

### 2.3. Direct Provisional Fixed Partial Denture

In the direct technique, patient's prepared teeth and the gingival tissues directly provide the tissue surface form eliminating all the intermediate laboratory procedures. This is convenient when assistant training and the office laboratory facilities are inadequate for efficiently producing an indirect restoration. However the direct technique has significant disadvantages like potential tissue trauma from the polymerizing resin and inherently poorer marginal fit. Therefore, the routine use of directly formed interim restoration is not recommended when indirect techniques are feasible.

**Procedure:**

1. Before the tooth preparation, place an acrylic tooth in place of the missing tooth and make an alginate impression or a putty index.
2. Prepare the patient's teeth in the usual manner.
3. Lubricate the prepared teeth and the adjacent gingival margins with petroleum jelly, and reseat the index or the alginate impression with provisional restorative material in the dough stage on the tissue surface of the impression.
4. Remove and reseat the restoration until it sets.
5. Finish, polish, and cement the restoration (Figure 3).

### 2.4. Alternative Techniques for Direct Technique

**(1) Acrylic Resin Block Technique for Direct Provisional Restoration**

A useful, though seldom employed, method for making provisional restorations is the acrylic resin block technique. It provides a means of fabricating the interim restoration without the use of diagnostic casts and laboratory processing costs. The technique requires knowledge of dental anatomy and the patience and artistic traits inherent in dentists.

**Procedure:**

- Tooth Preparation is carried out in a usual manner.
- Autopolymerizing acrylic resin of the suitable shade is mixed and allowed to set to a doughy consistency (the sheen of surface-free monomer has completely disappeared). After the abutments and surrounding gingiva have been lightly lubricated with petrolatum, the acrylic resin record is placed over the prepared abutments, and the patient is guided to closure in the centric occlusion position.
- The acrylic resin record is removed and replaced a few times during the curing process to minimize the effect of the exothermic heat on the abutments. After polymerization, the occlusal surface of the resin record is analyzed for anatomic design and may be marked with pencil as to cusp location and buccolingual width to facilitate carving the crown forms.
- Carbide burs and diamond stones are used to roughly develop contour and form of the provisional restoration.
- Since no impression matrix is used to carry the acrylic resin mix over the prepared teeth, the initial splint must be relined to assure adequate marginal adaptation and integrity. The inside of the crowns is relieved with a round carbide bur to provide space for the relining acrylic resin. The inner surfaces are moistened with monomer and filled with a fresh mix of acrylic resin. The splint is then replaced over the prepared abutments while the acrylic resin cures. The patient is again guided to closure in the centric occlusion position.
- The provisional restoration is carved to correct occlusal anatomy, crown contour, and embrasure form with burs, stones, and discs. This must be done with sufficient care and attention to detail so that it approximates the environment to be established by the final restoration. The provisional splint must be smooth and highly polished.
- The completed provisional restoration is now ready for placement with temporary cement. Zinc oxide and eugenol cements should be avoided, as they tend to soften the acrylic resin on contact and may weaken the restoration [3].

(2) Before starting to make a crown preparation, an irreversible hydrocolloid impression is made and immediately poured while waiting for the anesthetic to take effect. Following this an acrylic teeth is placed in the missing tooth region on the diagnostic cast and a shell matrix is custom made from mouthguard material. Lubricate the prepared tooth and adjacent teeth. Add just enough tooth-colored acrylic resin to fill only the prepared tooth space in the shell matrix and place the matrix over the teeth in the patient's mouth, pressing down on the adjacent teeth. Wait for the material to set, finish, polish, and cement the restoration. 

Alternatively, the restoration can be fabricated in a similar fashion outside the patient's mouth on the master cast after tooth preparation using the custom made shell matrix making it an indirect procedure [11].

(3) After contours of badly broken-down teeth are restored with wax, a preliminary alginate impression with a stock dentulous tray is made of the area to be prepared. Preferably, a complete-arch impression is obtained. The borders and septa are trimmed away from the set impression to facilitate reseating in the mouth. If a posterior fixed partial denture is to be made, a strip of irreversible hydrocolloid is removed from the edentulous ridge area to form a pontic in the completed temporary restoration. If an anterior fixed partial denture is to be made, then a denture tooth (or teeth) may be fixed in place with a small piece of soft rope wax prior to fabrication of the impression.

In an another technique, instead of replacing the missing tooth in temporary restoration in posterior quadrants, alginate impression can be scored in the form of a bar running across the edentulous region connecting the abutment teeth, thus producing a final restoration with crowns on the abutment teeth connected by a bar maintaining the integrity of the restoration. Instead of scoring a bar, a reverse pontic can also be scored in the alginate impression.

(4) In this technique, after removing the impression tray from the mouth, one should shorten the proximal projections of the impression material, and trim away the excess impression material palatally/lingually and buccally/facially to ensure complete reseating of the tray intraorally. Then, in this preoperative impression, grooves has to be created starting 1 mm buccally and lingually to the margin of the prepared tooth and continue towards the buccal and lingual flange areas to provide a pathway for the excess interim restorative material to escape [12].

(5) In another technique, a provisional removable partial denture which is often used to replace anterior teeth prior to fixed prosthodontic treatment is used as an aid in making a provisional fixed restoration. 

An irreversible hydrocolloid impression of the anterior segment of the provisional removable partial denture is made. Cold-cure acrylic resin of an appropriate shade is poured into the impression or placed into it with the powder-liquid method. The cured resin is removed from the impression as a block section of the anterior teeth and stored in water until needed. When the provisional fixed splint is being made, this block section of pontics is directly attached to the provisional crowns made for the abutment teeth. The block section of pontics may also be helpful if the provisional removable restoration is lost. The abutment teeth can then be prepared, individual provisional abutment crowns made, and the pontic section added. Alternatively, the unprepared abutment teeth can be acid etched and the block section of acrylic pontics directly attached to them with composite resin.

Alternatively, in an indirect way, an impression can be made with the existing removable partial denture in place, and this impression may be used to make the temporary restoration by placing it on the master cast that would be made after the tooth preparation.

(6) In any of these techniques, instead of building up the entire tooth with autopolymerizing resin, the acrylic tooth can be trimmed in the form of a labial veneer and the rest of the tooth built up with autopolymerizing resin. This tooth that has been trimmed in the shape of a veneer can be either used directly in the patient's mouth and rest of the tooth built up or can be used indirectly on a cast.

 (7) Using the existing prosthesis as a provisional restoration: when a cemented fixed prosthesis is to be removed for the reason of remaking it, damage to the prosthesis is of little concern. The important principle in such a case is to remove the prosthesis with minimum risk to the natural abutment teeth. It is possible to remove a cemented fixed prosthesis with little or no risk of damage to the abutment teeth by sectioning the prosthesis and expanding the retainer. Once removed, the prosthesis can be rebuilt to be used as a provisional or temporary prosthesis. The advantages of using the existing prosthesis are that the long-span fixed partial denture is stronger with metal reinforcement; the prosthesis incurs less occlusal wear with a metal or porcelain restoration versus an acrylic restoration; less time is required for fabricating a temporary restoration [13].

Alternatively, an impression of the existing fixed partial denture may be made before attempting its removal, and this impression may be used to make the temporary restoration by placing it on the master cast that would be made after the tooth preparation.

(8) In cases the patient presents with tooth preparation already being done and without a temporary, the following measures may be undertaken.
  - To build up the prepared tooth with the carding wax and place an acrylic tooth in the area of missing tooth and take an impression and use it to fabricate the temporary restoration.
  - To make the impression of the prepared tooth as it is and then score the impression in the form of reverse pontic and also in the area of the prepared tooth in an attempt to duplicate the unprepared tooth.
  - To use acrylic resin block technique forming direct provisional restoration.

(9) Provisional restoration for post and core restorations:
  - If custom made post and core is to be used, the post and core portion can be instantly built and temporary crown be fabricated on it.
  - If cast post is to be placed in the final restoration, the following measures may be taken.
    - A ball pin may be placed into the post space and an alginate over impression made that would pick up the ball pin and then the restoration fabricated on the cast.
    - Instead of placing the ball pin directly into the post space, it may be placed into the impression and the restoration fabricated.
    - In an alternative technique, a ball pin may be placed into the post space and the restoration fabricated intraorally using acrylic resin block technique. A tooth trimmed in the form of a labial veneer can also be used to serve the purpose.

## Figures and Tables

**Figure 3:**